# The clinical utility of nasal MRSA PCR as an antimicrobial stewardship tool to guide MRSA bacteraemia therapy in paediatrics: a retrospective study at a tertiary care centre

**DOI:** 10.1093/jacamr/dlag012

**Published:** 2026-02-17

**Authors:** Fahad Alrashed, Ebrahim Alsaadoon, Aeshah Alosaimi, Sameer Desai, Reem Almutairi, Bander Alrshaid, Sami Alhajjar, Ohoud Alyabes, Esam Albanyan, Mohammed Alsuhaibani, Suliman Aljumaah, Ibrahim Bin Hussain, Salem M Alghamdi

**Affiliations:** Paediatric Infectious Diseases Section, King Faisal Specialist Hospital & Research Centre, Riyadh, Saudi Arabia; Paediatric Infectious Diseases Section, King Faisal Specialist Hospital & Research Centre, Riyadh, Saudi Arabia; Infection Control and Hospital Epidemiology Department, King Faisal Specialist Hospital & Research Centre, Riyadh, Saudi Arabia; Biostatistics Epidemiology & Scientific Computing Department, King Faisal Specialist Hospital & Research Centre, Riyadh, Saudi Arabia; Biostatistics Epidemiology & Scientific Computing Department, King Faisal Specialist Hospital & Research Centre, Riyadh, Saudi Arabia; Pathology and Laboratory Medicine Department, King Faisal Specialist Hospital & Research Centre, Riyadh, Saudi Arabia; Paediatric Infectious Diseases Section, King Faisal Specialist Hospital & Research Centre, Riyadh, Saudi Arabia; Paediatric Infectious Diseases Section, King Faisal Specialist Hospital & Research Centre, Riyadh, Saudi Arabia; Paediatric Infectious Diseases Section, King Faisal Specialist Hospital & Research Centre, Riyadh, Saudi Arabia; Paediatric Infectious Diseases Section, King Faisal Specialist Hospital & Research Centre, Riyadh, Saudi Arabia; Paediatric Infectious Diseases Section, King Faisal Specialist Hospital & Research Centre, Riyadh, Saudi Arabia; Paediatric Infectious Diseases Section, King Faisal Specialist Hospital & Research Centre, Riyadh, Saudi Arabia; Paediatric Infectious Diseases Section, King Faisal Specialist Hospital & Research Centre, Riyadh, Saudi Arabia; Infection Control and Hospital Epidemiology Department, King Faisal Specialist Hospital & Research Centre, Riyadh, Saudi Arabia

## Abstract

**Objectives:**

Vancomycin is the main therapeutic choice for paediatric methicillin-resistant *Staphylococcus aureus* (MRSA) infections. However, its use is associated with many clinical challenges. This study aimed to investigate the use of MRSA polymerase chain reaction (PCR) as an antimicrobial stewardship tool to enhance therapeutic decision-making in paediatric MRSA bacteraemia.

**Methods:**

This retrospective cohort study was conducted at a tertiary healthcare centre from January 2021 to January 2024. It included paediatric patients who were screened for MRSA nasal colonization via PCR and had a blood culture obtained within 7 days of the MRSA PCR collection date. Clinical characteristics were compared between the positive and negative PCR groups. Positive and negative predictive values were determined. The association between mortality and anti-MRSA therapy was explored for patients with no confirmed MRSA infection.

**Results:**

A total of 1136 PCR tests were analysed with 161 positive results. The positive PCR group had a higher proportion of patients with intensive care unit admission, inotropic support and absolute neutrophil count >500. The positive PCR group had a higher proportion of patients who received anti-MRSA therapy, although no significant difference was observed in the therapy duration between the groups. The nasal MRSA PCR showed a positive predictive value (PPV) of 5.6% and a negative predictive value of 99.8%. When the analysis was restricted to patients with paediatric systemic inflammatory response syndrome, the PPV increased to 9%. Among patients who had negative PCR and culture results, a higher risk of mortality was observed for those using anti-MRSA therapy.

**Conclusion:**

Nasal MRSA PCR may serve as a valuable component of antimicrobial stewardship programmes to optimize the prescribing practices of anti-MRSA therapy.

## Introduction

In the 1960s, methicillin-resistant *Staphylococcus aureus* (MRSA) emerged owing to the presence of the mecA gene, which confers resistance to β-lactam antibiotics.^1–3^ Vancomycin became the main treatment choice for MRSA infections.^4,5^ However, numerous clinical challenges arose with the use of vancomycin, particularly in achieving appropriate concentrations in the paediatric age group. Furthermore, these challenges are accompanied by risks, such as renal impairment and histamine-induced reactions.^4,5^ Recent studies have reported an increase in cases of vancomycin-resistant *S. aureus*, highlighting the need for controlled antibiotic use and antimicrobial stewardship to manage resistance.^6–8^

Typically, vancomycin is used in combined therapy for hospital-associated infections to provide empiric coverage for MRSA. The Surviving Sepsis Campaign in children highlights the importance of empiric coverage against possible pathogens in cases of suspected bacteraemia. It also emphasizes the challenges of pathogen isolation in the paediatric age group via blood culture, resulting in unnecessary antibiotic exposure.^9^ At present, clear guidance on when to withhold or terminate vancomycin therapy is lacking.

Multiple systematic reviews have shown the high negative predictive value of nasal MRSA polymerase chain reaction (PCR) and its role in guiding vancomycin use for pneumonia. However, evidence of its utility in bacteraemia remains insufficient, particularly in the paediatric population.^10–12^ The primary objective of this study was to evaluate the use of nasal MRSA PCR as an antimicrobial stewardship tool to minimize patient exposure to anti-MRSA therapy by examining its predictive value in determining the likelihood of MRSA growth in blood cultures and its impact on bacteraemia management. The secondary objective was to compare the mortality rates between patients who received anti-MRSA therapy and those who did not in the absence of a confirmed MRSA infection (positive blood culture).

## Methods

### Study design and setting

This retrospective cohort study was conducted at the King Faisal Specialist Hospital and Research Center in Riyadh, Saudi Arabia, a tertiary care academic institution specialized in organ and haematopoietic stem cell transplantation with a 960-bed capacity. The study period was from January 2021 to January 2024. During this time, nasal MRSA screening via PCR was routinely performed upon admission for patients with a history of hospitalization within the past 6 months, those admitted to critical care units or transferred after 7 days from the previous test, and those undergoing cardiothoracic surgery. Positive cases were placed in contact isolation according to infection control protocols, with decolonization performed on a case-by-case basis.

### Study population

Paediatric patients below 15 years old were included if they had a nasal MRSA PCR swab and a blood culture obtained within 7 days of the MRSA PCR swab date. The 7-day cutoff is based on evidence indicating that the colonization status for admitted patients can change in as few as 7 days.^13^ Patients who failed to meet the aforementioned criteria or whose blood culture was obtained more than 24 h after the initiation of anti-MRSA therapy were excluded.

### Microbiological method

Nasal MRSA PCR swabs were collected using the Cepheid Sample Collection Device (Part No. 900-0370 Dual Swab in Liquid Stuart Media, Cepheid, USA) by registered nurse staff from the anterior nares of the patients. PCR testing was conducted using the Xpert SA Nasal Complete cartridge (GXSACOMP-CE-10, Cepheid, USA) utilizing GeneXpert^®^ Infinity-80 (XPERTISE_SWKIT_6.8, Cepheid, USA). Upon receiving the swab, one of the dual swabs was broken inside the buffer tube that was provided for each cartridge by the manufacturer, then vortexed for 15 s and it is lifted to sit for approximately 1 min. The tube content was transferred into the cartridge using a sterile transfer pipette and then loaded into the machine. The system is a closed-system PCR. The generation of the result took 45–50 min.

Our methodology for processing paediatric blood cultures has been described elsewhere.^14^

### Data collection

Data were collected from the patient’s medical records, including patient demographics, date and result of the nasal MRSA swab PCR, date and result of blood culture obtained within the specified timeframe, and information on whether the patient met the paediatric systemic inflammatory response syndrome (pSIRS) criteria as defined in the 2005 International Paediatric Sepsis Consensus Conference statement.^15^ MRSA colonization was defined as a positive result of nasal swab PCR, whereas MRSA bacteraemia was defined as the growth of methicillin-resistant *S. aureus* in a blood culture. Immunocompromised status was defined as the presence of a primary immunodeficiency, human immunodeficiency virus infection, malignancy, history of bone marrow or solid organ transplantation, or receipt of chemotherapy or radiotherapy. Patients receiving systemic immunosuppressive medications, including biologic agents, or high-dose corticosteroids (>2 mg/kg/day for more than 14 days), were also classified as immunocompromised. Data on bacteraemia management included the type of anti-MRSA agent used and the therapy duration (defined as the number of days the patient received at least one dose of anti-MRSA therapy). Furthermore, death that occurred within 30 days of a negative blood culture was recorded for patients with negative PCR results to estimate its association with anti-MRSA therapy.

### Ethical consideration

The study was approved by the ethics committee of KFSHRC (2241110). The requirement for informed consent was waived owing to the retrospective nature of the study.

### Statistical analysis

The samples were characterized using descriptive statistics, including means, standard deviations and frequencies. Patient demographics, clinical characteristics and outcomes were compared between the positive and negative PCR groups for the total sample and for the immunocompromised and immunocompetent subgroups. In the comparison, the Wilcoxon rank-sum test, Pearson’s chi-squared test or Fisher’s exact test was used for significance testing as appropriate. The positive predictive value (PPV) and negative predictive value (NPV) of MRSA PCR for MRSA bacteraemia were calculated first for the total sample and subgroups and then for those with pSIRS. The 95% confidence intervals (CIs) of the predictive values were generated using the exact method.^16^ A logistic regression model was used to explore the association between anti-MRSA use (Yes versus No) and 30-day mortality in a subgroup of patients with negative MRSA PCR and MRSA culture. This analysis was conducted to determine whether the absence of MRSA infection, as indicated by negative PCR and culture results, could inform clinical decisions regarding the use of anti-MRSA therapy and how such decisions may impact patient outcomes, particularly in terms of mortality. To account for repeated testing per patient and make valid inferences from the model, the generalized estimating equation was employed. The results were expressed as odds ratios (ORs) and their corresponding 95% CIs. The variables included in the model were pSIRS status, inotrope use, sex, age, immune status (only in the model for the total sample) and level of care (ICU versus ward). These variables were selected on the basis of their potential impact on mortality, reflecting disease severity and host vulnerability.

## Results

A total of 1136 MRSA PCR screening events were identified and included in our analysis. Among immunocompetent patients, 282 PCRs were negative and 78 were positive. In the immunocompromised group, 693 were negative and 83 were positive. This corresponded to 975 negative and 161 positive PCR results overall, with immunocompromised patients comprising 68% of the cohort. Table 1 presents a comparison of the demographic and clinical characteristics stratified by MRSA PCR results. The analysis revealed that the positive PCR group was significantly younger across all subgroups, with an average age difference of 1.2 years. Furthermore, a higher proportion of the positive PCR group in the total (57% versus 46%) and the immunocompromised subgroup (49% versus 37%) received care in the intensive care unit (ICU) compared with the negative PCR group. Inotrope use was more frequent in patients with positive PCR across all subgroups, with a significant difference in the overall sample (*P* = 0.007) and the immunocompromised subgroup (*P* = 0.019). Furthermore, the positive PCR immunocompetent subgroup had a higher proportion of patients with pSIRS than the negative group (73% versus 57%, *P* = 0.011). The majority of the included patients had an absolute neutrophil count >500, with a significant difference between the positive and negative PCR groups in the total sample (91% versus 79%) and the immunocompromised subgroup (83% versus 72%). Death within 30 days from the culture date was not significantly different between the negative and positive PCR groups in either the total sample or subgroups (*P*  ≥  0.2).

**Table 1.** Patient demographics and other characteristics stratified by MRSA PCR results

Characteristic	Total (*n* = 1136)			Immunocompetent (*n* = 360)			Immunocompromised (*n* = 776)		
	Negative (*n* = 975)	Positive (*n* = 161)	*P*-value	Negative (*n* = 282)	Positive (*n* = 78)	*P*-value	Negative (*n* = 693)	Positive (*n* = 83)	*P*-value
Age, mean (SD)	5.7 (3.7)	4.4 (3.9)	**<0.001**	5 (3.95)	3.4 (3.9)	**<0.001**	6. (3.5)	5.3 (3.7)	**0.040**
Sex			0.7			0.3			0.10
Female	475 (49)	76 (47)		141 (50)	44 (56)		334 (48)	32 (39)	
Male	500 (51)	85 (53)		141 (50)	34 (44)		359 (52)	51 (61)	
Immune status			**<0.001**			—			—
Immunocompetent	282 (29)	78 (48)		—	—	—	—	—	—
Immunocompromise	693 (71)	83 (52)		—	—	—	—	—	—
Level of care			**0.011**			0.5			**0.027**
ICU	452 (46)	92 (57)		196 (70)	51 (65)		256 (37)	41 (49)	
Ward	523 (54)	69 (43)		86 (30)	27 (35)		437 (63)	42 (51)	
No. of PCR tests, mean (SD)	1.1 (0.5)	1.1 (0.3)	0.5	1.1 (0.3)	1.1 (0.4)	0.3	1.2 (0.5)	1.1 (0.2)	0.2
Inotrope use	63 (6.5)	20 (12)	**0.007**	29 (10)	10 (13)	0.5	34 (4.9)	10 (12)	**0.019**
pSIRS	559 (57)	100 (62)	0.3	161 (57)	57 (73)	**0.011**	398 (57)	43 (52)	0.3
ANC > 500	775 (79)	147 (91)	**<0.001**	279 (99)	78 (100)	>0.9	496 (72)	69 (83)	**0.025**
Death within 30 days from the culture date	25 (2.6)	2 (1.2)	0.4	4 (1.4)	2 (2.6)	0.6	21 (3.0)	0 (0)	0.2

Data are expressed as mean (SD) or *n* (%).

ANC, absolute neutrophil count; ICU, intensive care unit; pSIRS, the paediatric systemic inflammatory response syndrome. Bold *P*-values indicate statistical significant results (<0.05).

The differences between the positive and negative PCR groups in terms of antibiotic administration for MRSA bacteraemia management are summarized in Table 2. The use of anti-MRSA antibiotics was significantly higher in the positive PCR than in the negative PCR group in the total sample (61% versus 48%, *P* = 0.001) and the immunocompetent subgroup (73% versus 58%, *P* = 0.014). However, the therapy duration did not significantly differ between the negative and positive PCR groups in either the total sample or the subgroups, with *P*-values ranging from 0.10 to 0.08. As regards the type of anti-MRSA agent used, vancomycin was the most common, followed by linezolid and clindamycin in the immunocompromised and immunocompetent subgroups, respectively, with no significant difference between the positive and negative PCR groups.

**Table 2.** Summary of bacteraemia management (antibiotic administration) stratified by MRSA PCR results

Characteristic	Total (1136)			Immunocompetent (360)			Immunocompromised (776)		
	Negative (975)	Positive (161)	*P*-value	Negative (282)	Positive (78)	*P*-value	Negative (693)	Positive (83)	*P* value
Anti-MRSA used	464 (48)	99 (61)	**0.001**	163 (58)	57 (73)	**0.014**	301 (43)	42 (51)	0.2
Duration of therapy, days, mean (SD)	10 (15)	12 (11)	0.10	9 (9)	11 (11)	0.4	10 (18)	13 (12)	0.080
Type of anti-MRSA			0.071			0.2			0.2
Clindamycin	17 (3.7)	8 (8.1)		11 (6.7)	8 (14)		6 (2.0)	0 (0)	
Linezolid	40 (8.6)	3 (3.0)		5 (3.1)	1 (1.8)		35 (12)	2 (4.8)	
Switched between agents	50 (11)	11 (11)		17 (10)	2 (3.5)		33 (11)	9 (21)	
Vancomycin	356 (77)	77 (78)		130 (80)	46 (81)		226 (75)	31 (74)	

Data are expressed as *n* (%); mean (SD). Bold *P*-values indicate statistical significant results (<0.05).

Table 3 presents the predictive values of MRSA PCR for MRSA blood culture results stratified by immune status and the presence of pSIRS. For the total sample and the immunocompromised subgroup, the MRSA PCR NPVs of the MRSA cultures were 99.79% and 99.71%, respectively. When the analysis was limited to those who met the pSIRS criteria, these values remained high at 99.64% and 99.5%, respectively. In the immunocompetent subgroup, all individuals with negative PCR had negative MRSA cultures, resulting in a 100% NPV in the presence and absence of pSIRS. In contrast, the MRSA PCR PPV for the MRSA culture was generally low. However, the results improved in the presence of pSIRS, particularly in the total sample and the immunocompromised subgroup (9% and 11%, respectively).

**Table 3.** Predictive values of MRSA PCR for MRSA blood culture

Patient group	Patient No.	NPV (95% CI), %	PPV (95% CI), %
Total	1136	99.79 (99.3–100.0)	5.59 (2.6–10.3)
Total with SIRS	659	99.64 (98.7–100.0)	9 (4.2–16.4)
Immunocompetent	360	100 (98.7–100.0)	5.13 (1.4–12.6)
Immunocompetent with SIRS	218	100 (97.7–100.0)	7 (1.9–17)
Immunocompromised	776	99.71 (99.0–100.0)	6.02 (2.0–13.5)
Immunocompromised with SIRS	441	99.50 (98.2–99.9)	11.63 (3.9–25.1)

Data on the association between anti-MRSA therapy and 30-day mortality in a subgroup of patients with negative MRSA screening and culture results are presented in Table 4. Anti-MRSA therapy was associated with an increased risk of mortality in the total sample (OR = 2.3, *P* = 0.044) and in the immunocompromised subgroup (OR = 2.6, *P* = 0.036) in a model adjusted for pSIRS, inotrope use, age, sex, immune status and level of care. The presence of pSIRS was significantly associated with increased mortality risk, particularly in the total sample and the immunocompromised subgroup. In contrast, the immunocompetent subgroup demonstrated reduced odds of mortality with anti-MRSA therapy, although this finding did not achieve statistical significance. The factors significantly associated with mortality in the immunocompetent subgroup were inotrope use (OR = 21.4, *P* = 0.004) and ICU admission (*P* < 0.001), with the latter being shared across all subgroups.

**Table 4.** Relationship between anti-MRSA therapy and 30-day mortality in patients with negative MRSA screening and culture results

Characteristic	Total (973)			Immunocompetent (282)			Immunocompromised (691)		
	OR	95% CI	*P*-value	OR	95% CI	*P*-value	OR	95% CI	*P* value
Anti-MRSA used									
No	—	—		—	—		—	—	
Yes	2.3	1.0, 5.4	**0.044**	0.5	0.2, 1.4	0.2	2.6	1.1, 6.1	**0.036**
pSIRS									
No	—	—		—	—		—	—	
Yes	2.1	1, 4.2	**0.050**	3.00	0.3, 28.5	0.3	2.5	1.1, 5.5	**0.026**
Inotrope use									
No	—	—		—	—		—	—	
Yes	1.6	0.5, 5.3	0.4	21.4	2.7, 170	**0.004**	0.4	0.1, 2.3	0.3
Age	0.9	0.8, 1	0.088	0.9	0.7, 1	0.12	0.9	0.8, 1.1	0.3
Sex									
Female	—	—		—	—		—	—	
Male	2	0.8, 5.0	0.15	0.94	0.1, 8	>0.9	2.4	0.8, 6.8	0.11
Immune status									
Immunocompetent	—	—		—	—	—	—	—	—
Immunocompromise	3.9	1.4, 11	**0.012**	—	—	—	—	—	—
Level of care									
ICU	—	—		—	—		—	—	
Ward	0.4	0.1, 0.9	**0.036**	0.00	0.00, 0.00	**<0.001**	0.3	0.1, 0.9	**0.030**

Bold *P*-values indicate statistical significant results (<0.05).

## Discussion

This study aimed to evaluate the utility of nasal MRSA PCR in predicting the growth of MRSA in blood culture and its impact on MRSA bacteraemia management, particularly in the context of immunocompromised versus immunocompetent patients. In patients who met pSIRS criteria, the NPVs were 99.5% for immunocompromised and 100% for immunocompetent subjects. These findings can play a pivotal role in antimicrobial stewardship programmes aimed at guiding vancomycin use and minimizing unnecessary exposure to anti-MRSA therapy. However, because predictive values are influenced by disease prevalence, our results should be interpreted in the context of an MRSA prevalence ranging from 32% to 42% of S. aureus infections (Supplementary material, available as Supplementary data at *JAC-AMR* Online).

Other studies have also obtained a high NPV for nasal MRSA PCR in predicting MRSA infection in clinical settings, particularly pneumonia, although paediatric-specific data are limited.^10–12^ Lexi *et al*. conducted a retrospective analysis of 3860 encounters with 2889 unique participants in critically ill paediatric patients, reporting an NPV of 99.8% across all culture sites.^5^ Another study involving 505 participants targeted the utility of such an intervention in respiratory infections, obtaining similar NPV (99.8%).^17^

As part of stewardship efforts and capitalization on the high NPV of nasal MRSA PCR, certain groups have integrated PCR results into their workflows to inform decisions regarding anti-MRSA therapy. A retrospective study reported that implementing a pharmacist-initiated protocol for MRSA PCR testing in patients with pneumonia significantly reduced the mean duration of vancomycin use (2.5 versus 1.4 days) in the PCR group.^18^ Another study employed a similar pharmacy-driven approach to guide anti-MRSA therapy in patients with COVID-19 with suspected secondary pulmonary bacterial infections and reported an 81% reduction in anti-MRSA therapy use, mainly due to early discontinuation or withholding initiation.^19^ Recently, an additional RCT protocol has been published to test the use of PCR screening in optimizing the empiric use of vancomycin therapy for pneumonia to provide high-quality evidence on the potential benefits of these results in reducing unnecessary antibiotic use.^20^ These findings suggest a promising impact when PCR results are incorporated into the decision-making process for anti-MRSA therapy. They also highlight the need for further investigation into how screening tests can best guide vancomycin use in different clinical contexts, particularly when its use as empiric therapy is more common, as in the case of suspected bacteraemia.

Our study also evaluated the association between anti-MRSA therapy and 30-day mortality in patients with negative MRSA screening and blood culture results. Within this group, anti-MRSA therapy was associated with a significant increase in mortality risk in the total sample.

When a subgroup analysis was conducted, the association persisted among immunocompromised patients, suggesting that receipt of anti-MRSA therapy in this subgroup in the absence of confirmed MRSA infection may reflect underlying conditions that predispose to worse outcomes. The significant associations between mortality, pSIRS and ICU admission support this interpretation and highlight the importance of considering the overall clinical picture when evaluating this relationship. In contrast, lower odds of mortality were observed in the immunocompetent subgroup receiving anti-MRSA therapy, although this trend was not statistically significant. The absence of a significant association in this subgroup suggests that withholding initiation or early discontinuation of treatment in the absence of confirmed infection may not adversely impact patient outcomes. However, future studies are warranted to further investigate this association and provide high-quality conclusions.

This study has several strengths. First is the large sample size and the use of a standardized protocol for MRSA screening, which enhances the reliability and robustness of the findings. Second, the distinction between immunocompromised and immunocompetent patients provides clinically relevant insights, particularly in the clinical decisions related to the management of MRSA bacteraemia in different patient populations. However, this study also has some limitations that need to be acknowledged. First, the retrospective nature and reliance on data from a single healthcare centre might introduce potential bias. Second, the unique patient population at our centre, a specialized organ and haematopoietic stem cell transplantation centre, combined with an MRSA prevalence of 32%–42%, may constrain the generalizability of our results to the general population. Future studies could benefit from including a more diverse population and differing MRSA prevalence to enhance the external validity of their findings.

To conclude, this study demonstrates the potential advantage of incorporating MRSA PCR results into clinical workflows to optimize antibiotic use, particularly in the context of suspected bloodstream infections. It highlights the high NPV of MRSA PCR in ruling out MRSA bacteraemia, which was 99.5% in immunocompromised patients and 100% in immunocompetent patients who met the pSIRS criteria. Withholding initiation or early termination of antibiotics, when appropriate in clinical settings, can enhance clinical practice, leading to better antimicrobial utilization and improved patient outcomes.

## Supplementary Material

dlag012_Supplementary_Data
