# Supplementary material for: The clinical utility of nasal MRSA PCR as an antimicrobial stewardship tool to guide MRSA bacteraemia therapy in paediatrics: a retrospective study at a tertiary care centre
Source: JAC Antimicrob Resist. 2026 Feb 17;8(1):dlag012. doi: 10.1093/jacamr/dlag012 (PMC12910373; doi:10.1093/jacamr/dlag012)
Supplement: dlag012_Supplementary_Data [file dlag012_supplementary_data.pdf]

The clinical utility of nasal MRSA PCR as an antimicrobial stewardship tool to guide MRSA bacteraemia therapy in paediatrics: a retrospective study at a tertiary care centre

Fahad ALRASHED[1], Ebrahim ALSAADOON[1], Aeshah ALOSAIMI[2], Sameer DESAI[3], Reem ALMUTAIRI[3], Bander ALRSHAID[4], Sami ALHAJJAR[1], Ohoud ALYABES[1], Esam ALBANYAN[1], Mohammed ALSUHAIBANI[1], Suliman ALJUMAAH[1], Ibrahim BIN HUSSAIN[1], Salem M. ALGHAMDI[1,2]*

1. *Paediatric Infectious Diseases Section, King Faisal Specialist Hospital & Research Centre, Riyadh, Saudi Arabia*
2. *Infection Control and Hospital Epidemiology Department, King Faisal Specialist Hospital & Research Centre, Riyadh, Saudi Arabia*
3. *Biostatistics Epidemiology & Scientific Computing Department, King Faisal Specialist Hospital & Research Centre, Riyadh, Saudi Arabia*
4. *Pathology and Laboratory Medicine Department, King Faisal Specialist Hospital & Research Centre, Riyadh, Saudi Arabia*

-Supplementary material-

Percentage of MRSA among S. aureus isolates as presented at our facility antibiogram

Year	Organism	Count	% of MRSA*
2021	Staphylococcus aureus	859	32
2022	Staphylococcus aureus	899	42
2023	Staphylococcus aureus	979	39

*Isolates resistant to Cefoxitin which is used as surrogate for Oxacillin
